# Retinoic Acid-Induced 2 (RAI2) Is a Novel Antagonist of Wnt/β-Catenin Signaling Pathway and Potential Biomarker of Chemosensitivity in Colorectal Cancer

**DOI:** 10.3389/fonc.2022.805290

**Published:** 2022-03-01

**Authors:** Weitao Zhang, Lu Kong, Hongbin Zhu, Decong Sun, Quanli Han, Bin Yan, Zhi Cui, Weiwei Zhang, Shurong Zhang, Xindan Kang, Guanghai Dai, Niansong Qian, Wenji Yan

**Affiliations:** ^1^ Department of Oncology, The First Medical Center, Chinese People’s Liberation Army (PLA) General Hospital, Beijing, China; ^2^ Cancer Center, Beijing Tongren Hospital, Capital Medical University, Beijing, China; ^3^ Medical Department, Chinese People’s Liberation Army (PLA) General Hospital, Beijing, China; ^4^ Department of Gastroenterology and Hepatology, Chinese People’s Liberation Army (PLA) NO.983 Hospital, Tianjin, China; ^5^ Department of Gastroenterology and Hepatology, The First Medical Center, Chinese People’s Liberation Army (PLA) General Hospital, Beijing, China

**Keywords:** RAI2, Wnt/β-catenin signaling, colorectal cancer, chemosensitivity, stem cell-like properties

## Abstract

**Objective:**

Aberrant activation of Wnt/β-catenin signaling contributes to the maintenance of cancer stem cells and chemoresistance in colorectal cancer (CRC). Retinoic acid-induced 2 (RAI2) was proved to be a tumor suppressor in CRC in our previous report. In this study, the role of RAI2 in Wnt/β-catenin signaling was further investigated.

**Methods:**

As a transcriptional co-regulator, C-terminal Binding Protein 2 (CtBP2) was reported to be involved in Wnt signaling in multiple and complex ways. The correlation of RAI2 and CtBP2 in CRC was analyzed by TCGA dataset, and the interaction between RAI2 and CtBP2 was explored by co-immunoprecipitation (Co-IP) in CRC cells. The effect of RAI2 on the activity of Wnt signaling and the location of β-catenin was detected by Dual-Luciferase reporter assay and Immunofluorescence respectively. Western blotting analysis was performed to detect the expression of target genes involved in Wnt signaling. Sphere formation assay was employed to detect the effect of RAI2 on stem cell like properties. Cell viability assay was used to detect the chemosensitivity of cells before and after transfection of RAI2.

**Results:**

The interaction between RAI2 and CtBP2 was confirmed by Co-IP in CRC cells. Besides, the negative correlation of RAI2 and CtBP2 in CRC was found by analyzing the TCGA dataset. Re-expression of RAI2 in human colon cancer cells (HCT116 and LoVo) suppressed the fluorescent activity of Wnt signaling, increased the phosphorylation and inhibited nuclear translocation of β-catenin, with down-regulation of target genes like c-Myc, CyclinD1, ASCL2, and LGR5. In contrast, the mutated RAI2, which can’t interact with CtBP2, has no above effects. We observed low expression of RAI2 in 33.89% (101/298) of CRC patients, which was significantly associated with reduced phosphorylation of β-catenin (r=0.8866, P<0.0001), poor 5-year relapse-free survival (RFS) (P = 0.0029) and overall survival (OS) (P = 0.0102). Restoration of RAI2 in HCT116 and LoVo cells inhibited stem cell-like properties of CRC cells and increased chemosensitivity of these cells to oxaliplatin and fluorouracil.

**Conclusion:**

Low expression of RAI2 can serve as an independent poor prognostic marker. RAI2 inhibits Wnt signaling by interacting with or down-regulating CtBP2, resulting in repression of stem cell-like properties and increased chemosensitivity of CRC cells.

## Introduction

Colorectal cancer (CRC) is the third most commonly diagnosed cancer worldwide, with more than 1.9 million new cases and almost 935,000 deaths in 2020 (1). 5-fluorouracil has always been the basis of all chemotherapy schedules, used alone or combined with other agents. The addition of oxaliplatin and irinotecan to fluorouracil-based treatment has increased response rate (RR) and overall survival (OS) (2–4). However, the chemoresistance is a great obstacle for colorectal cancer treatment. New strategies to improve chemotherapy sensitivity may help improve the treatment and prognosis of colorectal cancer.

The canonical Wnt (Wnt/β-catenin) signaling controls lots of biological processes, including cell fate determination, cell proliferation, and stem cell maintenance (5). 90% of the colorectal tumors have a mutation in one of the key regulatory factors of the Wnt/β-catenin signaling pathway, resulting in aberrant activation of this pathway, and up to 80% of tumors exhibit nuclear accumulation of β-catenin (6–8). Over 70% of colon cancers contain a mutation in adenomatous polyposis coli (APC) genes, which are associated with the earliest stages of colorectal carcinogenesis (9, 10). C-terminal Binding Proteins (CtBP1/2) are evolutionarily conserved metazoan transcriptional co-regulators. Transcriptional co-regulation by CtBPs has been found to play an important role in several diseases, including human cancer (11–14). CtBPs intersects with Wnt signaling in multiple and complex ways as revealed by work in both model organisms and human cancer cells and tumors (15, 16). In mutant APC setting, CtBPs promotes oligomerization of truncated APC by binding to 15 amino acid-repeats (17), facilitating the release of β-catenin into the nucleus and activation of downstream oncogenic β-catenin transcriptional targets, such as Cyclin D1. Additionally, CtBPs may directly coactivate TCF4/LEF at key target promoters (c-Myc, LGR5) to promote cancer stem cell self-renewal (18). And CtBP2 haplo-insufficiency could rescue polyposis induced by mutation of APC in the min mouse model of the human Familial Adenomatous Polyposis (19). Recently, it was proved that CtBP2 reduced chemosensitivity of non-small cell lung cancer cells to cis-diamminedichloroplatinum (CDDP) *via* promoting the Wnt/β-catenin pathway (20).

Retinoic acid-induced 2 (RAI2) was initially described as a retinoic acid-inducible gene, which has been assumed to be involved in development (21, 22). The role of RAI2 in human cancer had not been recognized until Werner et al. reported that early hematogenous dissemination of breast cancer cells, particularly in hormone receptor-positive tumors, is mediated by RAI2 (23). Further, our previous study demonstrated that RAI2 served as a tumor suppressor in colorectal cancer by inhibiting proliferation and promoting apoptosis of CRC cells, and the expression of RAI2 was down-regulated by promoter region methylation (24). And recently, it was found that RAI2, as a target gene of circular RNA RBPMS, played a key role in inhibiting bladder cancer cell growth (25). Interestingly, It was reported that RAI2 has two highly orthologically conserved sequences (ALDLS) in the internal region (amino acids 316–320 and 342–346 in human sequence) of the RAI2 protein. RAI2 can interact with CtBP2 through ALDLS domains and inhibit the transcriptional repression effect of CtBP2 on target genes (23).

In this study, we identified that RAI2 is a novel antagonist of Wnt/β-catenin signaling pathway and a potential biomarker of chemosensitivity in colorectal cancer. We demonstrated that RAI2 inhibits Wnt signaling by interacting with or down-regulating CtBP2, resulting in the inhibition of stem cell-like properties of CRC cells. Moreover, RAI2 increased the chemosensitivity of colorectal cancer cells to oxaliplatin and fluorouracil.

## Materials and Methods

### Primary Human Colorectal Cancer Samples and Cell Lines

A total of 298 cases of primary colorectal cancer were surgically resected. All tissues were collected from the Chinese PLA General Hospital according to the approved guidelines of the Chinese PLA General Hospital’s Institutional Review Board. In addition, three colorectal cancer cell lines (LoVo, HCT116, and SW620) were included in this study. All colorectal cancer cell lines were purchased from the Chinese Academy of Medical Science (CAMS) and Peking Union Medical College (PUMC), which were previously established from primary colorectal cancer. These cell lines were maintained in 90% RPMI 1640 (Invitrogen, CA, USA) supplemented with 10% fetal bovine serum. For the analysis of inducible expression of RAI2 by retinoic acid, colorectal cancer cell lines were split to low density (25% confluence) 12 h before treatment. In the detection of retinoic acid inducible effect on RAI2, Cells were treated with retinoic acid (RA) (Sigma, St. Louis, MO) at different concentrations of 0, 1, 5, 10μM in the growth medium. After that, the protein was extracted for Western blot detection of RAI2 expression.

### RNA Isolation and Semi-Quantitative RT-PCR

Total RNA was extracted using Trizol reagent; cDNA was synthesized according to the manufacturer’s instructions (Invitrogen, CA, USA). Actin was used as control. The RAI2 PCR primer sequences were as follows: 5′-ATGGCAATGCCACCTACGTCATG-3′ (forward) and 5′- GATACGGCACCAAGAGGGTGGC -3′ (reverse); CtBP2 PCR primer sequences were as follows: 5′- CAAGGCCTTTGGATTCAGCGTC -3′ (forward) and 5′- AGGGCTTGTGCTAAGGCTTTCT -3′ (reverse); the Actin PCR primer sequences were as follows: 5′- CTCCATCCTGGCCTCGCTGT -3′ (forward) and 5′- GCTGTCACCTTCACCGTTCC-3′ (reverse). Each experiment was repeated three times.

### Immunohistochemistry (IHC)

IHC staining was performed on 4-μm thick serial sections derived from formaldehyde-fixed paraffin blocks. Rabbit polyclonal antibody against RAI2 (Abcam, ST. Louis, MO) was diluted at 1:30, Rabbit anti-phospho-β-catenin (Thr41) (BIOSS, Beijing, China) was diluted at 1:1200, Rabbit anti- achaete-scute complex homolog 2 (ASCL2) (BIOSS, Beijing, China) was diluted at 1:200 and Rabbit anti-Leucine-rich repeat-containing G-protein coupled receptor 5 (LGR5) (Abcam, ST. Louis, MO) was diluted at 1:200. IHC was performed and the staining was evaluated as described previously (24), and samples were divided into 2 groups: high RAI2 expression group (score>3) and low RAI2 expression group (score ≤ 3).

### Plasmid Construction and Transfection

The expression vectors for RAI2 were subcloned into Plenti6 lentivirus expression vector, and RAI2 expression lentiviral or empty vectors were packaged using ViraPower™ Lentiviral Packaging Mix (Invitrogen, CA, USA) to infect LoVo and HCT116 cells to establish stable expression cells. The infected cells were selected by blasticidin (Invitrogen, CA, USA) at a concentration of 10 μg/ml. Lipofectamine 3000 (Invitrogen, CA, USA) was used for plasmid transfection. For transient transfection, the RAI2 CDS region was cloned into the pcDNA3.1 (+) plasmid (Era Biotech, Shanghai, China). To generate a RAI2 protein incapable of CtBP2 binding, we constructed the mutated RAI2 expression vector (RAI2-M), in which the binding sites, two conserved sequences (ALDLS) in RAI2 protein (amino acids 316–320 and 342–346 in human sequence), were mutated to ^316^ ALDAA ^320^ and ^342^ ALDAA ^346^ (23). All constructs were confirmed by sequencing.

### shRNA-Mediated Knockdown of Gene Expression

Four shRNA molecules were designed to target all four transcripts of RAI2 and constructed into pGPU6/GFP/Neo vector (GenePharma, Shanghai, China). These shRNAs were then transfected into SW620 cells according to the manufacturer’s instructions. The target sequences in the RAI2 gene were as follows: shRNA-1 (5′-GCTGTGCTCCAGAATTTGTTT-3′), shRNA-2 (5′-GCCACACGGTCATTAAGATGG-3′), shRNA-3 (5′-GGGAAGAGTCCATGGGAAATG-3′), and shRNA-4 (5′-GAAATACACATGCTCCCAATC-3′). The most effective construct, shRNA-2, was selected used in the follow-up experiment.

### Co-Immunoprecipitation

Three cell lines were used in this experiment, including LoVo, HCT116 and SW620. And pcDNA3.1(+) expression plasmids containing either the wild-type RAI2 (labeled with RAI2) or mutated RAI2 cDNA sequence (labeled with RAI2-M) were employed in this experiment, with pcDNA3.1(+) empty vector as control (labeled with vector). LoVo or HCT116 cells were transfected with 4 μg of above RAI2/RAI2-M/vector expression plasmids using Lipofectamine 3000 (Invitrogen, CA, USA). The following experiment was performed using Pierce Co-Immunoprecipitation(Co-IP) Kit (#26149,Thermo Scientific™, MA, USA) according to the manufacture’s instruction. IgG group was used as a negative control. Extracted cell lysates before adding antibodies (input) were also used as a control for target proteins, Actin, or GAPDH detection.

### Western Blot

Protein preparation and Western blot were performed as described previously (24). The antibodies for Western blot analysis were as follows: rabbit anti-RAI2 (#97857, Cell signaling technology, MA, USA, 1:1000), rabbit anti-phospho-β-catenin (ab27798, Abcam, Cambridge, UK, 1:500), rabbit anti-CtBP2 (ab128871, Abcam, Cambridge, UK, 1:10000), rabbit anti-β-catenin (ab32572, Abcam, Cambridge, UK, 1:5000), rabbit anti-CyclinD1 (ab134175, Abcam, Cambridge, UK, 1:10000), rabbit anti-c-Myc (ab39688, Abcam, Cambridge, UK, 1:500), rabbit anti ASCL2 (TA313480, ORIGENE, Rockville, US, 1:1000), rabbit anti LGR5 (ab199335, Abcam, Cambridge, UK, 1:1000), Anti-SOX2 (ab137385, Abcam, Cambridge, UK, 1:1000), Anti-CD133 [EPR20980-104] (ab216323, Abcam, Cambridge, UK, 1:1000), Anti-APC [EP701Y] (ab40778, Abcam, Cambridge, UK, 1:2000), and Anti-Oct4 antibody [GT486] (ab184665, Abcam, Cambridge, UK, 1:1000). And rabbit anti-GAPDH (ab181602, Abcam, Cambridge, UK, 1:10000) or anti-Actin (Cell Signaling Technology, Danvers, USA, 1:1000) was used as a control. Each experiment was repeated three times.

### Dual-Luciferase Reporter Assay

LoVo and HCT116 cells were seeded at 5 × 10^3^ cells/well in 96-well culture plates 24 h before transfection. To examine transcriptional activity driven by TCF/LEF, the above cells were transfected with 20 ng/well Topflash/Fopflash reporter vector (TCF/LEF–responsive reporter) and 2 ng/well pRL-TK. And 20ng/well pCI-neo-β-catenin-wt expressing vector, which expresses wild-type β-catenin was used to activate the reporter gene. Then, 200 ng/well of RAI2 was transfected into cells together with the Topflash/Fopflash reporter vector, pRL-TK control vector and pCI-neo-β-catenin-wt was used to evaluate the regulative effect of RAI2 on the Wnt signaling pathway. Forty-eight hours after transfection, relative luciferase activities were measured with the Dual-Luciferase Reporter Assay System (Promega, Madison, USA) according to the manufacturer’s protocol. In addition, LoVo and HCT116 cells, with or without RAI2 transfected, were treated with 20 mM LiCl (Sigma, St. Louis, MO) for 36hs before luciferase activity detection, and another two groups of LoVo and HCT116 cells, with or without RAI2 transfected, were treated with 10 µM XAV939 (MedChemExpress, Shanghai, China) for 36hs before luciferase activity detection. For each experiment, the luciferase reporter assay was performed three times.

### Immunofluorescence

Cells were grown on glass culture slides (BD Biosciences, San Jose, USA) and fixed with 4% cold methanol at -20°C for 10 min. Subsequently, cells were blocked with 10% goat serum for 1 h and incubated with primary antibodies against β-catenin (ab32572, Abcam, Cambridge, UK, 1:250) or RAI2 (PA5-62305 Invitrogen, CA, USA, 1:500) at 4°C for 1 h and then incubated with fluorescent labeled secondary antibodies for 1h at room temperature. After being counterstained with DAPI (Invitrogen, CA, USA), the slide was observed under a confocal microscope (Zeiss).

### Cell Sorting

LoVo and HCT116 cells were analyzed by fluorescence-activating cell sorting (FACS; BD bioscience). The cells were harvested using 0.05% trypsin (Gibco, CA, USA), washed twice with DPBS supplemented with 5% FBS and resuspended in DPBS supplemented with 10% FBS with mouse anti-human CD133/1 antibody (Miltenyi Biotec, Bergisch Gladbach, Germany) for 30 min at 4°C. The cells were washed twice with pre-cooled DPBS and centrifuged at 1200 rpm at 4°C and incubated in DPBS supplemented with 10% FBS with goat anti-mouse Alexa Fluor^®^ 488 for 30 min at 4°C in dark. After washing with DPBS twice, cells were sorted by FACS. CD133-negative and CD133-positive CRC cells were collected for further experiments, and they were cultured in RPMI 1640 supplemented with 10% FBS with 1% of penicillin/streptomycin.

### Sphere Formation Assay

In total, 1×10^3^ cells were seeded in 6-well ultra-low cluster plates (Corning, NY, USA) for 10 days. Spheres were cultured in RPMI 1640 serum-free medium (Invitrogen, CA, USA) supplemented with 2% B27 (Invitrogen, CA, USA), 20 ng/ml EGF, 20 ng/ml bFGF, 0.4% BSA, and 5 μg/ml insulin (Sigma, Beijing, China).

### Chemosensitivity Detection Assay

LoVo/HCT116 cells were treated with a concentration gradient of anticancer drugs before and following re-expression of RAI2. The anticancer drugs, oxaliplatin (L−OHP) (Sigma, Beijing, China) and 5−fluorouracil (5−FU) (Princeton, NJ, USA) were tested in six dilutions respectively. The concentrations of L−OHP for treatment were 0.00, 1.00, 2.00, 4.00, 8.00, 16.00μM in LoVo/HCT116 cells and 0.00, 2.00, 4.00, 8.00, 16.00, 32.00μM in SW620 cells. The concentration of 5−FU for treatment was 0.00, 6.25, 12.50, 25.00, 50.00, 100.00μM in LoVo/HCT116/SW620 cells. The cells were seeded at a density of 3x10^4^ cells/well on 96−well plates in RPMI−1640 medium, each well was repeated in triplicate. The following incubation overnight at 37˚C, the medium growth was replaced by a fresh medium containing the test drug. All the cells were treated for 48 h. At the end of the treatment, cell viability was measured by the MTT assay (KeyGEN Biotech, Nanjing, China). Absorbance was measured on a microplate reader (Thermo Multiskan MK3, MA, USA) at a wavelength of 490 nm. IC50 was defined as the concentration of L-OHP/5-FU for 50% inhibition of cell growth. Each experiment was repeated three times.

### Statistical Analysis

The RNA sequencing (RNA-Seq) data for RAI2 and CtBP2 gene expression in the CRC dataset were downloaded from The Cancer Genome Atlas (TCGA) (http://xena.ucsc.edu/). Statistical analysis was performed using SPSS 17.0 software. Chi-square or Fisher’s exact tests were used to evaluate the relationship between RAI2 expression status and clinical pathological characteristics. The two-tailed independent samples t-test was applied to determine the statistical significance of the differences between the two experimental groups. Spearman rank correlation test was used to determine the correlation between two groups of ordinal data. Survival rates were calculated by the Kaplan-Meier method, and the differences in survival curves were evaluated using the log-rank test. Cox proportional hazards models were fit to determine independent associations of RAI2's low expression with the 5-year OS and 5-year relapse-free survival (RFS) outcomes. Two-sided tests were used to determine the significance, and P < 0.05 was considered to be statistically significant.

## Results

### RAI2 Interacts With CtBP2 and Down-Regulates the Expression of CtBP2 in Colorectal Cancer Cells

**Figure 1:**
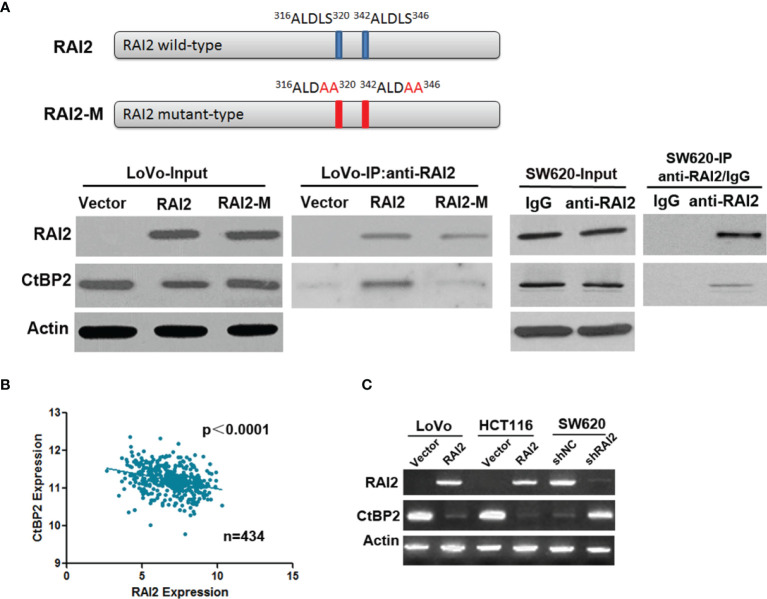
RAI2 expression is negatively correlated with that of CtBP2. **(A)** Upper panel: A schematic of RAI2 domain organization and the mutant sites of mutant type RAI2 (RAI2-M); lower panel: Coimmunoprecipitation (Co-IP) analysis of RAI2 and CtBP2 interaction by anti-RAI2 antibody using cell lysates from LoVo cells transfected with empty vector, RAI2 expression vector or RAI2 mutant vector. And Cell lysates from SW620 cells were also used for Co-IP detection to analyze the endogenous interaction between CtBP2 and RAI2 by RAI2 antibody, with IgG group as a negative control. Input: before any antibodies were added. **(B)** The reciprocal mRNA expression between CtBP2 and RAI2 in 434 cases of colorectal cancer tissues from the TCGA dataset. **(C)** RT-PCR detection of RAI2 and CtBP2 expression in LoVo/HCT116 cells with or without RAI2 re-expressed and in SW620 cells with or without RAI2 knocked down.

RAI2 was initially identified as a retinoic acid-induced gene (21). Firstly, we confirmed the induction effect of retinoic acid (RA) on RAI2 in LoVo and HCT116, in which basal RAI2 expression levels were low before RA treatment (**Supplementary Figure 1**). The primary RAI2 amino acid sequence has two highly orthologically conserved sequences (ALDLS) in the internal region (amino acids 316–320 and 342–346 in human sequence) of the RAI2 protein, and It was reported that RAI2 can interact with CtBP2 by ALDLS domain in breast cancer (23). In this study, to confirm the interaction between RAI2 and CtBP2 in colorectal cancer, we also employed the double mutant RAI2 with both sites were mutated to 316 ALDAA 320 and 342 ALDAA 346. The pull-down experiments demonstrated that when both sites were mutated, there was a marked decrease in coprecipitated CtBP2 in LoVo cells. To assess whether endogenous CtBP2 and RAI2 interact in CRC cells, we performed the same experiment in SW620 cells, in which basal RAI2 expression levels were high, and confirmed the interaction between RAI2 and CtBP2 (**Figure 1A**). Interestingly, in the input experiment group, we found that the expression level of CtBP2 in LoVo cells transfected with RAI2 expression vector was less than that of empty vector- or RAI2-M plasmid-transfected LoVo cells. In addition, by searching The Cancer Genome Atlas (TCGA) (http://xena.ucsc.edu/) databases and analyzing RNA sequencing (RNA-Seq) data in 434 cases of CRC samples, we found that the expression of CtBP2 was negatively correlated with the expression of RAI2 (p<0.0001) (**Figure 1B**). We further detected the expression of CtBP2 in LoVo and HCT116 cells with or without RAI2 restored by RT-PCR and found that the expression of CtBP2 was significantly decreased in LoVo and HCT116 cells with RAI2 re-expressed. And in SW620 cells, the expression of CtBP2 was significantly increased after RAI2 was knocked down (**Figure 1C**). The above results suggested that besides interacting with CtBP2, RAI2 can also down-regulate the expression of CtBP2 in colon cancer cells.

### RAI2 Inhibits Activation of Wnt/β-catenin Signaling in CRC Cells

**Figure 2:**
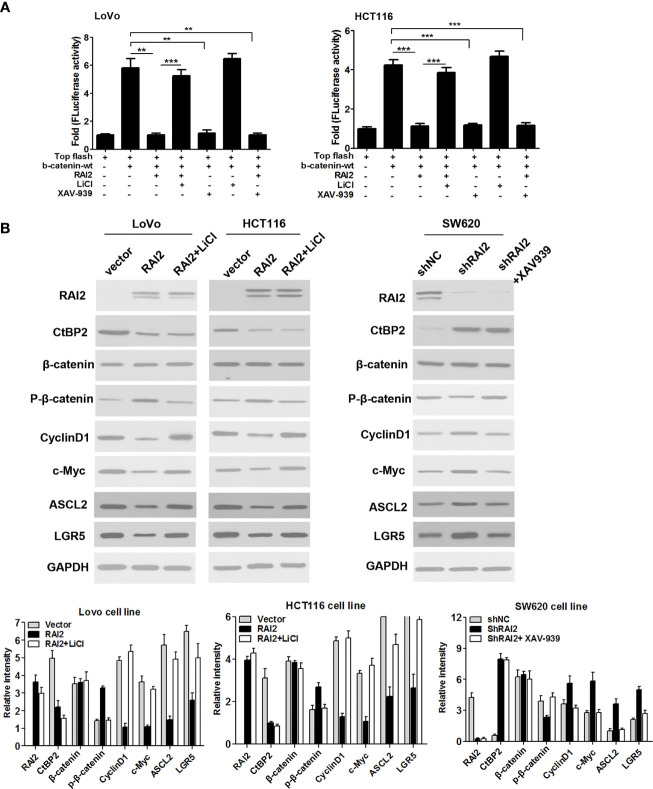
RAI2 suppressed activation of Wnt/β-catenin signaling pathway. **(A)** Dual-Luciferase reporter assay analyzed the effect of RAI2 plasmid transfection on wild-type β-catenin (β-catenin-wt)-induced activation of Wnt/β-catenin signaling in LoVo/HCT116 cells with or without LiCl/XAV939 addition (the concentrations of LiCl and XAV939 were 20mM and 10 µM respectively). LiCl and XAV939 were agonist and antagonist of Wnt/β-catenin signaling respectively. **(B)** Western blot analysis of RAI2, β-catenin, phosphorylated β-catenin (p-β-catenin) CtBP2 and the Wnt/β-catenin signaling target genes c-Myc, cylinD1, ASCL2, and LGR5 expression in LoVo/HCT116 cells with RAI2/empty vector transfection and RAI2-transfected cells with LiCl addition (20mM). The same experiment was also performed in shNC/shRAI2-transfected SW620 cells and shRAI2-transfected SW620 cells with XAV939 addition (10 µM). p-values: **<0.01; ***<0.001.

**Figure 3:**
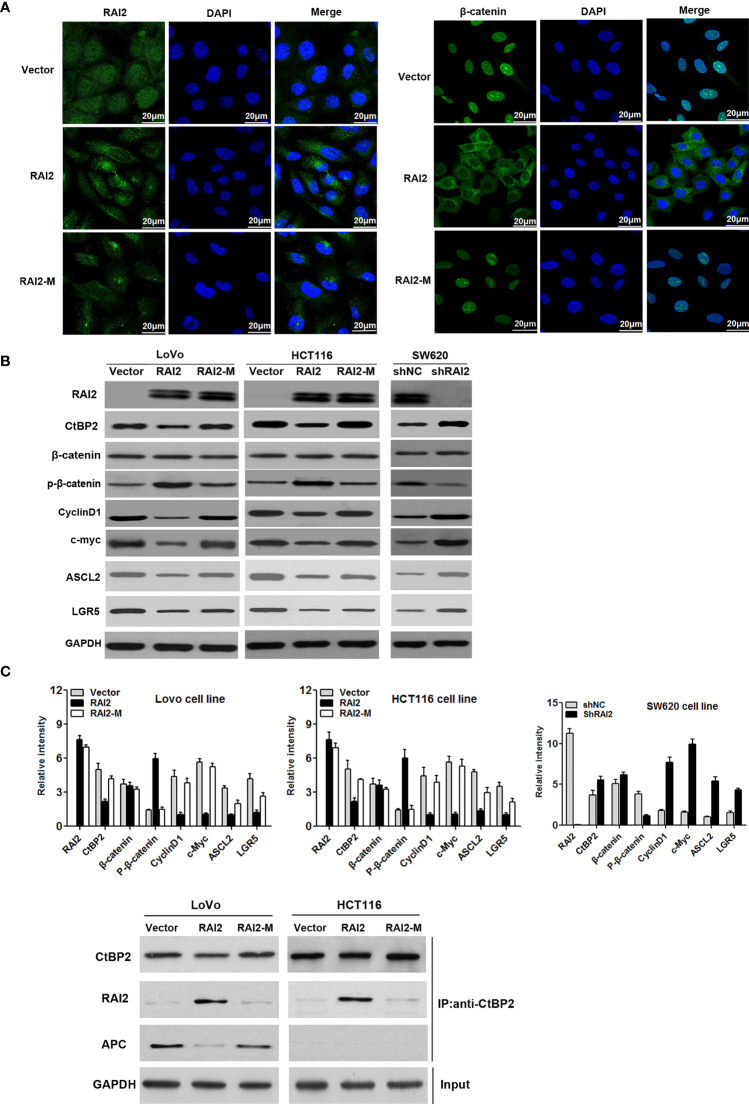
RAI2 inhibits the nuclear translocation of β-catenin and suppresses the expression of Wnt signaling target genes. **(A)** Immunofluorescence analysis of expression and localization of RAI2 and β-catenin in LoVo cells with empty vector/RAI2/RAI2-M (mutant type RAI2 plasmid) transfection. The left panel showed the expression and localization of RAI2 in LoVo cells before and after RAI2/RAI2-M transfection; the right panel showed the expression and localization of β-catenin in LoVo cells before and after RAI2/RAI2-M transfection. **(B)** Western blot analysis of RAI2, β-catenin, phosphorylated β-catenin (p-β-catenin), CtBP2, and the Wnt/β-catenin signaling target genes c-Myc, CylinD1, ASCL2, and LGR5 expression in LoVo/HCT116 cells with empty vector/RAI2/RAI2-M (mutant type RAI2 plasmid) transfection and SW620 cells with shNC/shRAI2 transfection. **(C)** Coimmunoprecipitation (Co-IP) analysis of CtBP2 and APC interaction by anti-CtBP2 antibody using cell lysates from LoVo and HCT116 cells transfected with vector, RAI2 expression vector or mutant type RAI2 vector (RAI2-M). Extracted cell lysates of LoVo and HCT116 cells before adding antibodies were used for GAPDH detection (Input).

Aberrant activation of Wnt/β-catenin signaling contributes to carcinogenesis and progression of colorectal cancer. CtBPs(CtBP1/2) was determined to be transcriptional co-repressor and oncogene in human cancer (26). CtBPs could promote the activation of Wnt/β-catenin by interacting with mutated APC or directly binding to TCF/LEF (16). To clarify the effect of RAI2 on Wnt signaling, we detected the luciferase activity of Wnt/β-catenin signaling before and after RAI2 transfection in colorectal cancer cells LoVo and HCT116, respectively. As shown in **Figure 2A**, the increased activity induced by wild-type β-catenin was significantly suppressed by RAI2 (P=0.0021 in LoVo, P=0.0004 in HCT116). In addition, LiCl and XAV939, the agonist and antagonist of Wnt/β-catenin signaling respectively, were employed to clarify the role of RAI2 in Wnt signaling. As expected, the agonist LiCl significantly enhanced the activity of Wnt signaling induced by wild-type β-catenin with RAI2 transfected (P-value was 0.0007 and 0.0006 respectively in LoVo and HCT116 cells), While, the antagonist XAV939 significantly inhibited Wnt/β-catenin signaling activity in both LoVo (P=0.0027) and HCT116 (P=0.0004) cells, and the inhibitory effect was similar or even more significant in RAI2-transfected CRC cells (P-value was 0.0021 and 0.0005 respectively in LoVo and HCT116 cells) (**Figure 2A**). We detected the effect of RAI2 on the expression of CtBP2, β-catenin, phosphorylated β-catenin, and the Wnt target genes, c-Myc, CyclinD1, ASCL2, and LGR5, by western blot. Significant reduction of CtBP2 and increase of phosphorylated β-catenin (p-β-catenin) was found in RAI2-transfected LoVo and HCT116 cells, with reduced expression of c-Myc, CyclinD1, ASCL2, and LGR5, the Wnt target genes. Besides, we found that the effects of RAI2 on expression of p-β-catenin, c-Myc, CyclinD1, ASCL2, and LGR5 could be eliminated by LiCl addition in RAI2-tranfected LoVo and HCT116 cells. While, no significant change of CtBP2 and β-catenin expression was found in RAI2-transfected LoVo and HCT116 cells with or without LiCl added. In contrast, increased expression of CtBP2 and reduced phosphorylated β-catenin (p-β-catenin) were found in SW620 cells with RAI2 knocked down (shRAI2-transfected SW620 cells) compared with control group (shNC-transfected SW620 cells), with upregulation of c-Myc, CyclinD1, ASCL2, and LGR5. While, except CtBP2, no significant change of above other proteins was found in RAI2-knocked down SW620 cells with XAV939 addition compared with shNC-transfected SW620 cells, which revealed that the effect of RAI2 on p-β-catenin and target genes of Wnt signaling was reversed by XAV939 (**Figure 2B**). The above results suggested that RAI2 served as an antagonist of Wnt/β-catenin signaling, while, the regulation of CtBP2 by RAI2 was not through Wnt signaling and RAI2 may inhibit Wnt/β-catenin by interacting with or down-regulating CtBP2, resulting in increased degradation of β-catenin. Furthermore, the localization of β-catenin in CRC cells with or without RAI2 restored was investigated by cell immunofluorescence assay, and Mutant type RAI2 (RAI2-M), in which ALDLS sequences were destroyed, was then employed in our study. Firstly, we confirmed the expression and localization of RAI2 in RAI2/RAI2-M-transfected LoVo cells, and found RAI2 expression both in cell cytoplasm and nucleus (**Figure 3A**, left panel). In LoVo cells without RAI2 restoration, β-catenin was mainly localized at the cell nucleus, which represents the activation of Wnt signaling. While, in LoVo cells with RAI2 re-expressed, β-catenin was mainly localized at cytoplasm. However, both in RAI2-M-transfected and vector control LoVo cells, β-catenin was localized at the cell nucleus (**Figure 3A**, right panel). Thus, we proposed that RAI2, not RAI2-M, inhibits the translocation of β-catenin from the cytoplasm to nucleus. consistently, Western blot detection found that the effect of RAI2 on phosphorylation of β-catenin, expression of CtBP2 and Wnt target genes c-Myc, CyclinD1, ASCL2, and LGR5 were lost when ALDLS sequences of RAI2 was destroyed. Thus, we found no significant change of the above proteins in LoVo/HCT116 cells transfected with RAI2-M compared with vector control (**Figure 3B**).

In addition, we further detected the effect of RAI2 on the interaction between CtBP2 and APC by Co-IP and found that APC was pulled down by CtBP2 antibody in vector control LoVo cells with mutant type APC, which was eliminated in RAI2-transfected LoVo cells. In contrast, APC was not pulled down by CtBP2 antibody in all three groups of HCT116 cells that have wild-type APC with vector, RAI2, or RAI2-M transfected (**Figure 3C**). It revealed that the inhibition of Wnt signaling by RAI2 in CRC with mutant APC may through destruction of interaction between CtBP2 and mutant APC, while in CRC with wild-type APC, the inhibition of Wnt signaling by RAI2 may involve a different mechanism, such as transcriptional down-regulation of CtBP2, an indirect approach.

These results suggested that the up-regulation of phosphorylated β-catenin depends on conserved sequences ALDLS of RAI2, by which RAI2 interacts with CtBP2. In addition, we can see the suppression of CtBP2 by RAI2, which was not seen in RAI2-M-transfected CRC cells. This result suggested that RAI2 may inhibit Wnt signaling by promoting the phosphorylation and subsequent degradation of β-catenin, and the effect of RAI2 on Wnt signaling may depend on the interaction between RAI2 and CtBP2 or the direct down-regulation of CtBP2 expression by RAI2.

### The Decreased Expression of RAI2 Is Related to Poor Prognosis of Colorectal Cancer Patients

**Figure 4:**
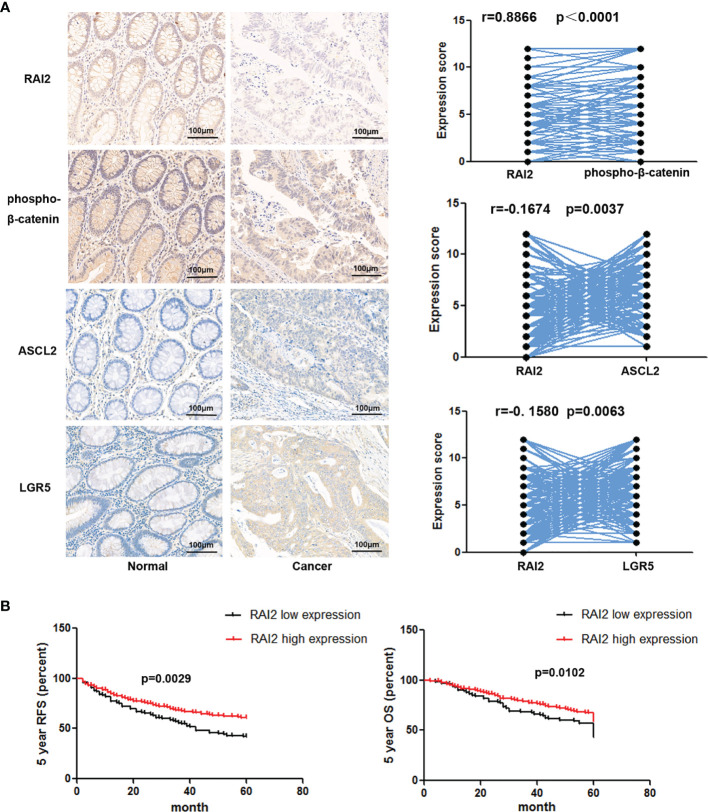
Low expression of RAI2 was associated with a low level of phosphorylation of β-catenin and poor outcome in CRC. **(A)** Left panel: IHC staining of RAI2, phosphorylated β-catenin (p-β-catenin), ASCL2, and LGR5 in CRC tissues; right panel: the correlation between RAI2 expression and expression of p-β-catenin, ASCL2, and LGR5 (Spearman rank correlation test). **(B)** Kaplan-Meier curves show the association of the 5-year overall survival (OS) rate and relapse-free survival (RFS) rate of colorectal cancer patients with the expression status of RAI2. Black, RAI2-low expressed colorectal cancer patients (n = 101, log-rank test); red, RAI2-high expressed colorectal cancer patients (n = 197, log-rank test).

We have reported the reduced expression of RAI2 regulated by promoter region methylation in colorectal cancer in our previous study. However, by searching The Cancer Genome Atlas (TCGA) (http://xena.ucsc.edu/) databases, the RAI2 expression data obtained by RNA sequencing (RNA-Seq) showed no association between RAI2 mRNA expression and 5-year overall survival (OS)/5-year relapse-free survival (RFS) (24). In this study, we detected the expression of RAI2 in 298 cases of colorectal cancer patients by IHC. Low expression of RAI2 was found in 33.89% (101/298) of the colorectal cancer samples, and the expression of RAI2 was positively correlated with phosphorylated β-catenin (r=0.8866, P<0.0001), which is further validation of the inhibitory effect of RAI2 on Wnt/β-catenin signaling. And we further detected the expression of ASCL2 and LGR5, two main Wnt target genes in CRC, in above 298 cases of CRC patients, and found that expression of RAI2 was negatively correlated with that of ASCL2 (r=-0.1674, P=0.0037) and LGR5 (r=-0.1580, P=0.0063) (**Figure 4A**). Though no association was found between RAI2 expression and clinical characteristics such as gender, TNM stage, lymph node metastasis, age, differentiation, tumor location, and size (P >0.05) (**Table 1**), Kaplan-Meier analysis indicated that low expression of RAI2 was significantly associated with poor 5-year RFS (P = 0.0029) and 5-year OS (P = 0.0102) (**Figure 4B**). According to univariate analysis, RAI2 expression and TNM stage were associated with both poor 5-year OS (all P < 0.05) and 5-year RFS (all P < 0.05, **Table 2**). In addition, age was associated with poor 5-year OS (P < 0.01), but not 5-year RFS (P >0.05). By multivariate analysis, RAI2 expression and TNM stage were associated with both poor 5-year OS and 5-year RFS (all P < 0.05, **Table 2**). Age was associated with poor 5-year OS (P < 0.01, **Table 2**). These results suggest that RAI2 expression was an independent prognostic marker for poor 5-year OS (P = 0.027, **Table 2**) and 5-year RFS (P = 0.022, **Table 2**).

**Table 1:** Clinical characteristics and RAI2 expression status of 298 patients with colorectal cancer.

Clinical parameter	RAI2 expression status			P value
	Low expression (N=101)		High expression (N=197)	
**Gender**				P=0.9468
Male	54	106		
Female	47	91		
**Age (year)**				P=0. 8436
>60	48	96		
≤60	53	101		
**Tumor location**				P=0.5764
Right-sided colon	23	39		
Left-sided colon	19	47		
Rectum	59	111		
**Tumor size (cm)**				
≤5	69	144		P=0.3871
>5	32	53		
**TNM stage**				P=0.8983
I-II	73	141		
III-IV	28	56		
**Differentiation**				P=0.5640
Middle-high	70	130		
Low	31	67		
**Lymph node metastasis**				P=0.1139
YES	95	174		
NO	6	23		
**Intravascular cancerous embolus**				P=0.4547
YES	16	25		
No	85	172		

P-values are obtained from chi-squared test.

**Table 2:** Analysis of RAI2 expression status with OS or RFS in colorectal cancer patients by Cox regression analysis.

Variables	OS				RFS			
	Univariate analysis		Multivariate analysis		Univariate analysis		Multivariate analysis	
	HR (95%CI)	P	HR(95%CI)	P	HR (95%CI)	P	HR(95%CI)	P
**RAI2 expression**	0.644	0.010*	0.683	0.027*	0.645	0.011*	0.671	0.022*
high vs low	(0.462-0.898)		(0.488-0.957)		(0.460-0.904)		(0.477-0.945)	
**Age (year)**	0.619	0.005**	0.560	0.001**	0.853	0.348	0.780	0.158
>60 vs **≤** 60	(0.444-0.863)		(0.397-0.789)		(0.612-1.189)		(0.553-1.101)	
**Gender**	1.021	0.900	0.950	0.764	1.010	0.953	0.950	0.767
Female vs Male	(0.735-1.420)		(0.678-1.331)		(0.724-1.409)		(0.675-1.337)	
**Tumor location**	1.026	0.900	1.174	0.467	1.034	0.872	1.250	0.322
distal colon or rectum vs proximal colon	(0.686-1.535)		(0.762-1.810)		(0.687-1.556)		(0.804-1.943)	
**Tumor size**	0.808	0.268	0.729	0.118	0.796	0.244	0.727	0.120
≤5 cm vs >5 cm	(0.554-1.178)		(0.490-1.083)		(0.543-1.168)		(0.486-1.087)	
**Differentiation**	1.055	0.765	1.091	0.630	0.998	0.990	1.038	0.839
Low vs High**/**middle	(0.744-1.495)		(0.766-1.553)		(0.700-1.423)		(0.725-1.486)	
**TNM-stage**	2.182	0.000***	2.439	0.000***	2.067	0.000***	2.237	0.000***
III/IV vs I**/**II	(1.558-3.055)		(1.708-3.483)		(1.464-2.918)		(1.550-3.228)	
**Pathologic N stage**	0.996	0.989	1.213	0.541	1.003	0.992	1.239	0.500
N1-2 vs N0	(0.538-1.843)		(0.653-2.253)		(0.542-1.856)		(0.665-2.311)	
**Intravascular cancerous embolus**	1.302	0.255	1.061	0.805	1.044	0.863	0.884	0.633
Yes vs No	(0.827-2.051)		(0.665-1.693)		(0.637-1.713)		(0.533-1.467)	

*P <0.05, **P < 0.01, ***P < 0.001.

### RAI2 Inhibits the Stem Cell-Like Properties of CRC Cells and Increases the Chemosensitivity of CRC Cells to Oxaliplatin and Fluorouracil

**Figure 5:**
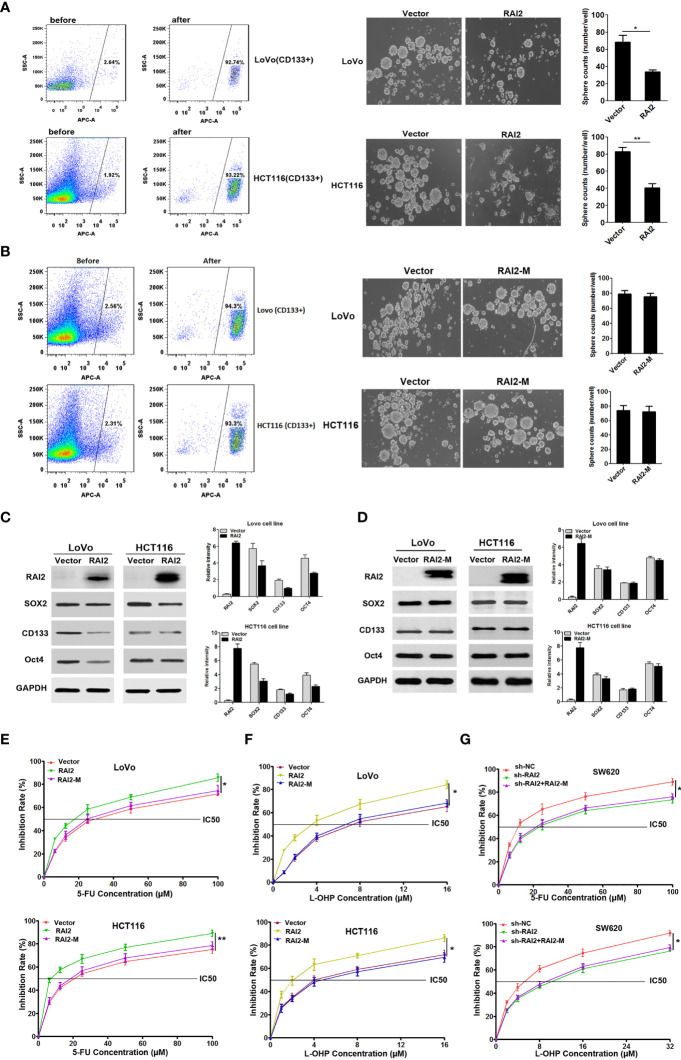
RAI2 inhibited CRC stem cell-like properties and increased the chemosensitivity of CRC cells to 5-FU and L-OHP. **(A)** Left panel: Representative images showing CD133-positive cell proportion before and after fluorescence-activating cell sorting by CD133 antibody in LoVo/HCT116 cells. Right panel: Representative images showing sphere-forming ability affected by RAI2 re-expression in LoVo/HCT116 cells. **(B)** Left panel: Representative images showing CD133-positive cell proportion before and after fluorescence-activating cell sorting by CD133 antibody in LoVo/HCT116 cells. Right panel: Representative images showing sphere-forming ability affected by RAI2-M re-expression in LoVo/HCT116 cells. **(C)** Western blot of CD133, SOX2 and OCT4 expression in RAI2 re-expressed or vector control cells. **(D)** Western blot of CD133, SOX2 and OCT4 expression in RAI2-M re-expressed or vector control cells. **(E)** Representative curves of growth inhibitory effects of 5-FU in LoVo/HCT116 cells with or without re-expression of RAI2/RAI2-M in different concentrations (0.00, 6.25, 12.50, 25.00, 50.00, 100.00μM). **(F)** Representative curves of growth inhibitory effects of L-OHP in LoVo/HCT116 cells with or without re-expression of RAI2/RAI2M in different concentrations (0.00, 1.00, 2.00, 4.00, 8.00, 16.00μM). **(G)** Growth inhibitory effect of 5-FU/L-OHP in SW620 cells with or without RAI2 knocked down and SW620 cells with both shRAI2 and RAI2-M transfected. The treatment concentrations of 5-FU in SW620 cells were (0.00, 6.25, 12.50, 25.00, 50.00, 100.00μM), and the treatment concentrations of L-OHP were (0.00, 2.00, 4.00, 8.00, 16.00, 32.00μM). The viability of cells was measured by MTT assay after 5-FU/L-OHP treatment for 48 hrs. p-values: *≤0.05; **<0.01.

Current findings support that the Wnt/β-catenin pathway plays a crucial role in the maintenance of stem cell-like properties and chemo-resistance in colorectal cancer (27). CD133 is a representative stem cell maker (28). In this study, we isolated CD133+ CRC cells (LoVo and HCT116) by flow cytometry (**Figure 5A**, left panel). Tumor sphere formation assay showed that CD133+ cells with RAI2 re-expressed formed fewer and smaller spheres than the control group (P=0.0285 in LoVo, P=0.0048 in HCT116) (**Figure 5A**, right panel), suggesting that RAI2 inhibited the self-renewal ability of CRC cells. while we found no significant difference in CD133+ cells with RAI2-M transfected compared to vector control group cells (P>0.5 in both LoVo and HCT116) (**Figure 5B**). Western blot assay showed that CD133+ LoVo and HCT116 cells with RAI2 re-expressed exhibited lower levels of stem cell makers like CD133, SOX2, and Oct4 than control LoVo and HCT116 cells (**Figure 5C**), while we found no significant difference between RAI2-M-transfected CD133+ cells and vector control group cells (**Figure 5D**). The above results revealed that re-expression of RAI2 inhibited the stem cell-like properties of CRC cells.

Chemoresistance is a hallmark of cancer stem cells (CSCs) (29). Thus, we detected the effect of RAI2 on chemosensitivity of colorectal cancer cells to oxaliplatin (L-OHP) and fluorouracil (5-FU) by MTT. We raised its level by RAI2 transfection in LoVo/HCT116 cells. To confirm the importance of the ALDLS sequence in RAI2 protein, we also performed the chemosensitivity assay in RAI2-M-transfected CRC cells, with the vector-transfected CRC cells as the control group. As shown in **Figure 5E**, The IC50 of 5-FU was decreased in RAI2-transfected LoVo cells (30.20 ± 6.09 μM versus 16.31 ± 2.89 μM, P=0.0234) and RAI2-transfected HCT116 cells (20.89 ± 4.23 μM versus 7.76 ± 1.66 μM, P=0.0075), while, we found no significant difference between RAI2-M-transfected CRC cells and vector control group in both LoVo and HCT116 (P>0.05). We gain similar results in L-OHP-treated cells, the IC50 of L-OHP was 7.90 ± 2.21μM versus 3.27 ± 0.37μM in LoVo cells with empty vetor and RAI2 transfected (P=0.0233), and 4.52 ± 1.25 μM versus 2.09 ± 0.56 μM (P=0.0373) in HCT116 cells with empty vetor and RAI2 transfected, while no significant difference was found in RAI2-M-transfected CRC cells controlled with vector group in both LoVo and HCT116 cells (P>0.05) (**Figure 5F**). On the contrary, reduced chemosensitivity was found in SW620 cells with RAI2 knocked down. The IC50 of 5-FU was 12.11 ± 1.90 μM versus 24.41 ± 6.51 μM (P=0.0349) in shNC- and shRAI2-transfected SW620 cells, and the IC50 of L-OHP was 4.76 ± 0.77 μM versus 8.74 ± 1.49 μM (P=0.0147) in shNC- and shRAI2-transfected SW620 cells, while, no significant difference was found between CRC cells with both shRAI2 and RAI2-M transfected and CRC cells with only shRAI2 transfected in both LoVo and HCT116 (P>0.05) (**Figure 5G**). The above results suggested that RAI2 increased the chemosensitivity of colorectal cancer cells to L-OHP and 5-FU. RAI2 may serve as a potential biomarker of chemosensitivity in colorectal cancer.

## Discussion

RAI2 is one of retinoic acid-induced genes, in this study, we have confirmed the inducible effect of retinoic acic on RAI2 (**Supplementary Figure 1**). As an important development-related gene, RAI2 has also been proved to serve as a key tumor suppressor in breast cancer and CRC (23, 24). CtBPs promotes a neoplastic phenotype by suppressing tumor suppressor gene expression and by facilitating upregulation of oncogenic factors (26). In vertebrates, CtBP family proteins are encoded by 2 paralogous genes, CtBP1 and CtBP2. These proteins display redundant functional and structural similarities (16, 30, 31). The transcription factor-binding domain in the N-terminus of CtBP is responsible for CtBP’s association with the majority of its known transcription factor and co-repressor partners (26, 30, 32). This occurs through CtBPs recognition of a consensus PxDLS (Pro-x-Asp-Leu-Ser; x is commonly a hydrophobic amino acid) peptide motif in its partner proteins (33). For example, histone deacetylases such as HDAC4, HDAC5 and HDAC7 contain a PxDLS motif through which CtBP proteins can interact to promote gene silencing (34). RAI2 has two highly orthologically conserved sequences (ALDLS) in the internal region (amino acids 316–320 and 342–346 in human sequence) of the RAI2 protein. It was reported that RAI2 can interact with CtBPs, especially CtBP2, through ALDLS domains and inhibit the transcriptional repression effect of CtBP2 on target genes in breast cancer (23). The interaction between RAI2 and CtBP2 was confirmed in this study in CRC cells (**Figure 1A**). Furthermore, by searching the TCGA dataset and analyzing RNA sequencing (RNA-Seq) data in 434 cases of CRC samples, we found a significant negative correlation between RAI2 and CtBP2 (p<0.0001) (**Figure 1B**), which was confirmed by RT-PCR in CRC cells (**Figure 1C**). It revealed that besides interacting with CtBP2, RAI2 may directly regulate the expression of CtBP2.

**Figure 6:**
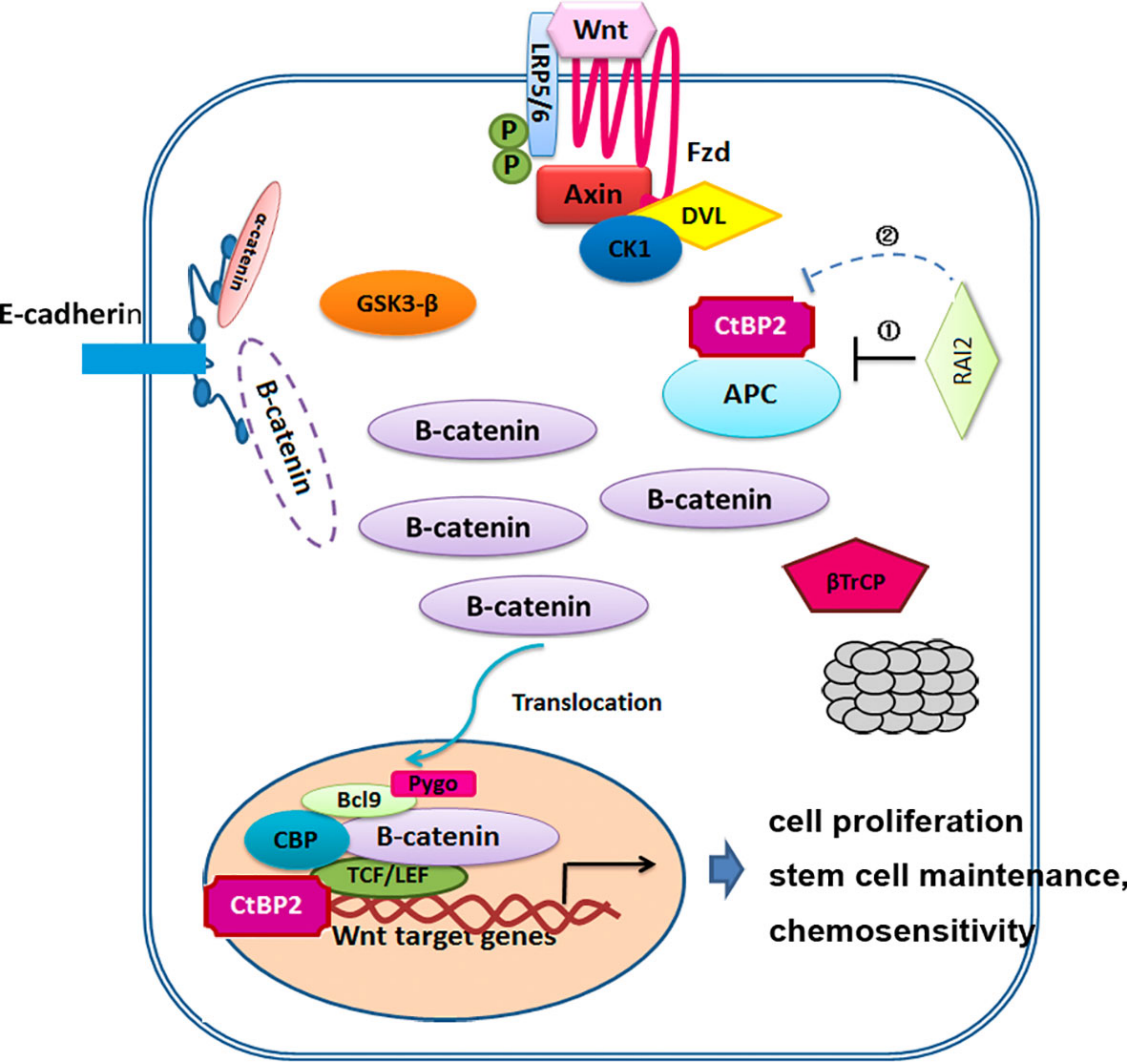
Mechanisms model for the regulation of RAI2 in Wnt/β-catenin signaling. ① RAI2 inhibits the interaction of CtBP2 and APC. ② RAI2 suppresses the expression of CtBP2.

Deregulation of Wnt/β-catenin signaling pathway plays an important role in human colorectal cancer. The key regulatory step of this pathway involves the phosphorylation, ubiquitination and degradation of β-catenin. These processes rely on a dedicated cytoplasmic destruction complex, which consists of the central scaffold protein Axin and three other core components, adenomatous polyposis coli (APC), the kinases glycogen synthase kinase-3 alpha/beta (GSK-3), and casein kinase-1 (CKI) (35). 90% of all colorectal tumors have a mutation in one of the key regulatory factors of the Wnt/β-catenin signaling pathway, resulting in the activation of the pathway, with up to 80% of tumors exhibiting nuclear accumulation of β-catenin (6–8). Over 70% of colon cancers contain a mutation in APC genes (9, 10). Mutated APC yielded an APC protein product that is truncated at the C-terminus. These products undergo oligomerization, resulting in their impaired ability to degrade β-catenin, resulting in β-catenin nuclear localization and interaction with TCF/LEF complex. In this mutant APC setting, CtBP promotes oligomerization of truncated APC by binding to 15 amino acid-repeats (17), facilitating the release of β-catenin into the nucleus and activation of downstream oncogenic β-catenin transcriptional targets, such as Cyclin D1. Additionally, CtBPs may directly coactivate TCF4/LEF at key target promoters (c-Myc, LGR5) to promote cancer stem cell self-renewal (18), indicating that CtBPs may activate Wnt signaling in cancer cells at multiple nodes-both by interacting with APC and several other genes that are associated with tumor initiation and growth, metastasis, and cancer stemness (15). It was reported that CtBP2 haplo-insufficiency could rescue polyposis induced by mutation of APC in the min mouse model of the human disease Familial Adenomatous Polyposis (19). In this study, we investigated the impact of RAI2 on Wnt signaling, and found that RAI2 could inhibit the Wnt signaling pathway, increase the phosphorylation of β-catenin, inhibit the nuclear translocation of β-catenin and down-regulate the target genes such as C-Myc, CyclinD1, ASCL2, and LGR5, while this effect depends on the ALDLS domain (**Figures 2** and **3**). It revealed that RAI2 may inhibit the activation of Wnt signaling by interacting with CtBP2. Furthermore, we confirmed the direct interaction between CtBP2 and APC in LoVo cells with mutant type APC, which could be eliminated when RAI2 was restored (**Figure 3C**). We proposed that the interaction of RAI2 and CtBP2 or down-regulation of CtBP2 by RAI2 may impair the binding of CtBP2 and mutant APC (**Figure 6**). And RAI2 may intersect the activation of TCF/LEF by CtBP2 and other coactivators, which need further investigation. Thus, RAI2 may regulate the Wnt signaling by down-regulating the expression of CtBP2 or direct binding to CtBP2, with more degradation, less nuclear translocation and inhibited binding to target genes of β-catenin.

In a recent study, multivariate analyses indicated that both RAI2 mRNA and RAI2 protein expression were independent risk factors for both disease-free survival (DFS) and breast-cancer-specific survival (BCSS) in breast cancer patients (36). In our previous study, the expression of RAI2 in 32 paired tissue samples was found reduced significantly in cancer tissue compared to the adjacent normal tissue by IHC detection. However, maybe because of the systematic set of cutoff value (around half samples were defined as RAI2 high expression and half samples were defined as RAI2 low expression), no association was found between RAI2 mRNA expression and 5-year OS (n = 333) or 5-year RFS (n =341) by RAI2 expression data analysis obtained from RNA sequencing (RNA-Seq) in TCGA dataset (24). In this study, we further detected the expression of RAI2 in 298 cases of CRC tissue samples by IHC, and found that low expression (score ≤3, 101/298) of RAI2 was significantly associated with reduced phosphorylation of β-catenin (r=0.8866, P<0.0001), poor 5-year OS (P = 0.0102), and 5-year RFS (P = 0.0029) (**Figures 4A, B**). In addition, multivariate analysis suggested that RAI2 low expression (score ≤3) was an independent prognostic marker for poor 5-year OS (P = 0.027) and 5-year RFS (P = 0.022 (**Table 2**). ASCL2 and LGR5 are two important target genes of Wnt signaling in colorectal cancer (37, 38). In our study, we found that RAI2 suppressed the expression of ASCL2 and LGR5 by Western blot in CRC cells, and we also found a negative correlation between RAI2 and ASCL2/LGR5 in 298 cases of cancer tissues from CRC patients by IHC detection (**Figure 4A**).

Wnt/β-catenin pathway plays a crucial role in chemoresistance in colorectal cancer. CtBP protein family was looked as an emerging oncogene and novel small molecule drug target (16). Depletion of CtBP2 inhibited, while its overexpression enhanced, CSC growth and self-renewal in colon cancer (18). A study reported that CtBP2 promotes proliferation and reduces drug sensitivity in non-small cell lung cancer *via* the Wnt/β-catenin pathway (20). Abdelaal MR. et al. reported that EC-synthetic retinoids can be effective, alone or in combinations, for a potential anticancer activity to colorectal cancer, with overexpression of E-cadherin, RAI2, and Werner (WRN) genes (39). Both 5-Aza-2′-deoxycytidine (DAC, a demethylation agent) and retinoic acid could induce the expression of RAI2 in colorectal cancer, which was proved in our previous (24) and supplementary data of this study. In addition, we found that RAI2 inhibits the stem cell-like properties of CRC cells and increases the chemosensitivity of colorectal cancer cells to oxaliplatin and fluorouracil (**Figure 5**), which revealed that RAI2 may serve as a potential biomarker of chemosensitivity in colorectal cancer. We proposed that maybe demethylating agent DAC, retinoic acid and EC-synthetic retinoids can serve as a novel choice in combination therapy of CRC patients with low expression of RAI2.

## Conclusion

This study reveals that low expression of RAI2 was an independent prognostic marker for poor 5-year OS and 5-year RFS. Our work provides evidence that silence or down-regulation of RAI2 in colorectal cancer may contribute to aberrant activation of Wnt signaling pathway, and consequently promote chemoresistance of CRC cells to chemotherapy drugs such as oxaliplatin and fluorouracil. Thus, RAI2-based detection may help to predict the chemosensitivity and provide a novel therapeutic target for the treatment of patients with CRC.

## Data Availability Statement

The original contributions presented in the study are included in the article/**Supplementary Material**. Further inquiries can be directed to the corresponding authors.

## Ethics Statement

The studies involving human participants were reviewed and approved by the ethics committee of PLAGH. The patients/participants provided their written informed consent to participate in this study.

## Author Contributions

Conceptualization, WY, GD, and NQ. Methodology, WY, WeitaoZ, and LK. Validation, WY, WeitaoZ, and LK. Formal analysis, WY, HZ, DS, and XK. Investigation, WY, LK, WeitaoZ, and WeiweiZ. Resources, WY, GD, and NQ. Data curation, WY, WeitaoZ, and LK. Writing-original draft preparation, WY, WeitaoZ, and LK. Writing-review and editing, QH, GD, and NQ. Visualization, WY, BY, SZ, and ZC. Supervision, QH, GD, and NQ. Project administration, GD and NQ. Funding acquisition, WY, GD, and NQ. All authors have read and agreed to the published version of the manuscript.

## Funding

This work was supported by grants from the National Natural Science Foundation of China (NSFC No.81802390, 31671298, 81672462), Natural Science Foundation of Beijing (BJNSF No.7202187), National Key Research and Development Program of China (No.2016YFC0905200, No.2016YFC0905302), Military Medicine Youth Special Project of PLA General Hospital (QNF19037), National Geriatrics Center Founding (NCRCG-PLAGH-2018002), Key Projects of Clinical Application and Promotion with Capital Characteristics (Z161100000516003).

## Conflict of Interest

The authors declare that the research was conducted in the absence of any commercial or financial relationships that could be construed as a potential conflict of interest.

## Publisher’s Note

All claims expressed in this article are solely those of the authors and do not necessarily represent those of their affiliated organizations, or those of the publisher, the editors and the reviewers. Any product that may be evaluated in this article, or claim that may be made by its manufacturer, is not guaranteed or endorsed by the publisher.

## Supplementary Material

Supplementary Figure 1 Retinoic acid induced the re-expression of RAI2 in CRC cells Western blot analysis of RAI2 expression in LoVo/HCT116 cells with retinoic acid treatment in different concentration (0, 1, 5, 10 uM). p-values: *≤0.05; ***<0.001.
