# Supplementary figures and images for: Retinoic Acid-Induced 2 (RAI2) Is a Novel Antagonist of Wnt/β-Catenin Signaling Pathway and Potential Biomarker of Chemosensitivity in Colorectal Cancer
Source: Front Oncol. 2022 Mar 1;12:805290. doi: 10.3389/fonc.2022.805290 (PMC8922473; doi:10.3389/fonc.2022.805290)

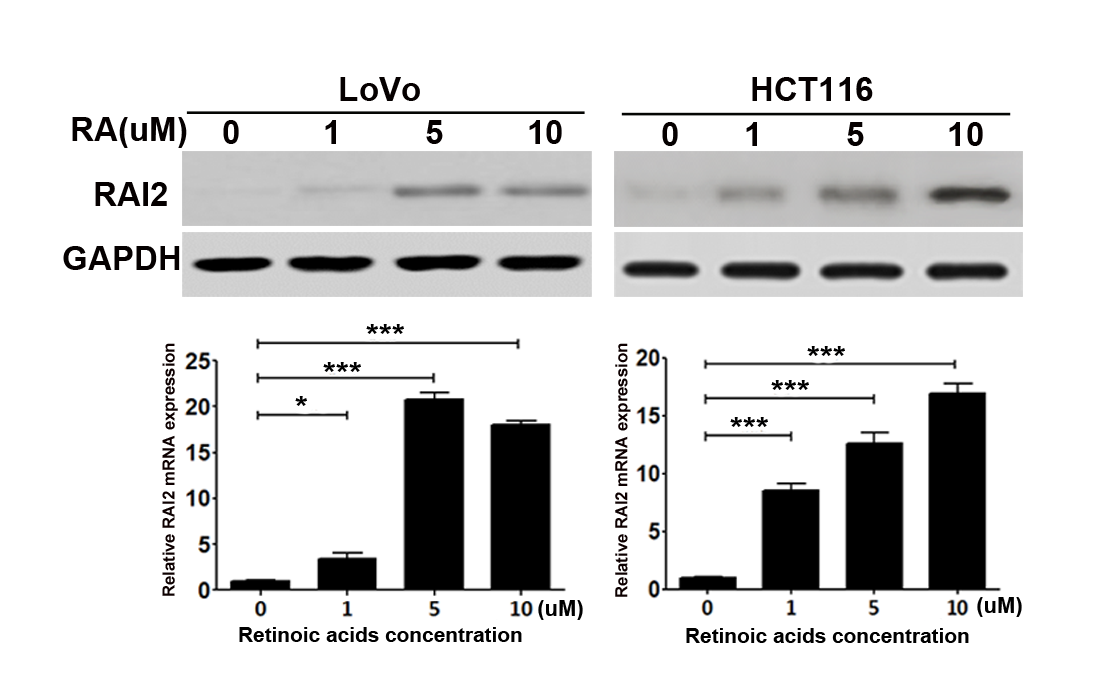

Supplement: Supplementary Figure 1 — Retinoic acid induced the re-expression of RAI2 in CRC cells Western blot analysis of RAI2 expression in LoVo/HCT116 cells with retinoic acid treatment in different concentration (0, 1, 5, 10 uM). p-values: *≤0.05; ***<0.001. [file Image_1.tif]
